# Shock Index on Admission Is Associated with Coronary Slow/No Reflow in Patients with Acute Myocardial Infarction Undergoing Emergent Percutaneous Coronary Intervention

**DOI:** 10.1155/2019/7873468

**Published:** 2019-07-25

**Authors:** Qingcheng Wang, Huimin Shen, Huijuan Mao, Fenghua Yu, Haiqing Wang, Jianlei Zheng

**Affiliations:** ^1^Department of Cardiology, Hangzhou Yuhang Hospital of Traditional Chinese Medicine, Yuhang 311106, Zhejiang, China; ^2^Department of Cardiology, Zhejiang Provincial People's Hospital, People's Hospital of Hangzhou Medical College, Hangzhou 310014, Zhejiang, China

## Abstract

**Objective:**

Coronary slow/no reflow is not rare after successfully undergoing primary percutaneous coronary intervention (PCI) in patients with acute myocardial infarction (AMI), and shock index (SI) is an important factor for adverse cardiovascular prognosis. In this study, we are to explore whether SI is associated with coronary slow/no reflow in patients with AMI following primary PCI.

**Methods:**

A total of 153 consecutive AMI patients undergoing primary PCI within 24 hours of symptom onset were included in this study. The participants were divided into normal flow group (n=124) and slow/no reflow group (n=29) according to cineangiograms recorded during the period of PCI. Cardiovascular risk factors, hematologic parameters, preoperative management of antithrombotic therapy, and baseline angiography were collected.

**Results:**

SI, plasma glucose, white blood cells (WBC) and neutrophil count, neutrophil to lymphocyte ratio (PLR), high sensitivity C-reactive protein (hs-CRP), probrain natriuretic peptide (pro-BNP), and Killip classification on admission and thrombus burden on initial angiography were significantly different between patients with and without slow/no reflow. Multivariate analysis revealed that SI≥0.66, thrombus burden, and plasma glucose on admission were independent predictors for coronary slow/no reflow. Preoperative management of tirofiban therapy improves initial thrombolysis in myocardial infarction (TIMI). However, it has no effect on prognosis of slow/no reflow.

**Conclusion:**

Our findings demonstrated that slow/no reflow in patients with AMI following primary PCI was more likely associated with SI≥0.66, thrombus burden, and plasma glucose on admission. SI as a predictor for coronary slow/no reflow should be further confirmed in the following more large-scale and prospective studies. The clinical registration number is ChiCTR1900024447.

## 1. Introduction

By the virtue of primary percutaneous coronary intervention (PCI) with stenting implantation and combination with the dual antiplatelet drugs and statins therapy, the short- and long-term mortality of patients with acute myocardial infarction (AMI) were significantly decreased [1–3]. However, the incidence of coronary slow/no reflow was as high as 20% to 30% according to previous literature, so patients continued to suffer from severe impairment of myocardial reperfusion due to coronary slow/no reflow after successful opening of infarct related artery (IRA) [4, 5]. The slow/no reflow commonly means the microvascular obstruction in distal coronary artery and is regarded as a predictor for adverse cardiovascular events [6, 7].

The pathogenesis of slow/no reflow phenomenon is sophisticated and has not been completely clarified up to now, but several hypotheses have been mentioned including distal microembolization of thrombus fragments, swelling of endothelial cells caused by ischemic and reperfusion injury, and microvascular spasm [8, 9]. In clinical practices, a great number of studies have been carried out to investigate the predictors for slow/no reflow phenomenon, and the results showed that thrombosis burden, reperfusion time, inflammatory factors, the ratio of stent size to vessel diameter, and prehospital treatment with tirofiban were potentially associated with slow/no reflow and clinical prognosis in patients with AMI following emergent PCI [10–15]. However, shock index (SI) was an important factor for main adverse cardiovascular events (MACEs) in patients with AMI [16, 17], but it has been rarely reported in slow/no reflow phenomenon.

In consideration of the phenomenon of slow/no reflow as a severe complication of catheterization laboratory and a pivotal indicator for clinical outcomes, the aim of this study is to explore whether SI, preoperative use of tirofiban, hematological paremeters, cardiovascular risk factors, and initial angiographic features effectively foresee angiographic slow/no reflow in patients with AMI after primary PCI.

## 2. Methods

### 2.1. Study Population

Between November 2016 and July 2018, a total of 153 consecutive patients with acute myocardial infarction included ST elevation myocardial infarction (STEMI) and non-ST elevation myocardial infarction (Non-STEMI). All the patients underwent PCI with at least one drug-eluting stent. The diagnosis of STEMI was based on the following criteria: typical ischemic chest pain that lasted more than 30 min and ST-segment elevation>1 mm in at least two contiguous leads or presumably new left bundle branch block on electrocardiogram combined with increased cardiac-specific biomarkers. In the absence of ST-segment elevation electrocardiogram, patients meeting the inclusion criteria were considered to have Non-STEMI. The patients with a history of recent surgery or trauma within the preceding 1 month and those with hematologic diseases, malignant tumors, febrile disorders, severe renal failure and hepatic dysfunction, and acute or chronic inflammatory disease on study entry were excluded. Besides, patients with AMI onset ≥ 24 hours were not enrolled either in this study. All patients were provided with written informed consent. The study protocol conformed to the ethical guidelines of the Declaration of Helsinki and was approved by the ethical committees of Zhejiang Provincial People's Hospital.

### 2.2. Study Protocol and Definitions

Coronary angiography and PCI were performed through radial access after symptom onset within 24 hours. The IRA was the only target of emergent PCI. Angiographic slow/no reflow during PCI was defined as TIMI flow grade ≤2 during the procedure without evidence of dissection, residual stenosis, distal embolism, or vasospasm. The TIMI flow grades were determined by the consensus of 2 intervention clinicians. Blood samples for measuring hematologic parameters, hs-CRP, pro-BNP, cardiac troponin I (cTNI), serum creatinine, blood urea nitrogen, and glucose were collected upon admission from a peripheral vessel in the emergency unit. Patients were given aspirin 300 mg and clopidogrel 300 mg as a loading dose before PCI. Then 100 mg of aspirin combined with 75 mg of clopidogrel once per day or 90 mg of ticagrelor twice per day was given after PCI. In addition, 10-15 ml tirofiban (100ml/5mg, Grand Pharmaceutical Co., Ltd., China) was given to patients with AMI onset ≥ 3 hours and accompanied with consistent angina in the emergency department. The angiographic morphologic features of burden thrombus in the IRA are scored in five degrees according to Gibson [18]: high burden thrombus formation if the TIMI thrombus was more than class 3. Multivessel coronary artery disease was defined as significant stenosis (coronary stenosis≥70%) of more than one epicardial coronary artery, including the culprit artery. Killip classification was introduced according to previous literature [19]. SI was defined as the ratio of heart rate to systolic blood pressure. In this study, the value of SI was measured in the emergency department. It was reported that SI≥0.66 was a strong predictor for MACEs in patients with AMI [16]. Hence, we further classified value of SI into ≥0.66 and <0.66. Clinical information of in-hospital outcomes included cardiac death for any reason, nonfatal reinfarction, ventricular tachycardia and/or ventricular fibrillation, and severe cardiac failure. Nonfatal reinfarction was defined as recurrent clinical angina symptoms with electrocardiogram changes compatible with MI or cTNI level at least twice the upper limit of normal range [20]. Severe cardiac failure was defined as congestive heart failure and/or cardiogenic shock that required treatment.

### 2.3. Statistical Analysis

Continuous variables are expressed as mean ± standard deviation (SD), and categorical data are presented as percentages. Differences in continuous variables between two groups were assessed by unpaired 2-tailed t-test. Categorical data and proportions were analyzed by chi-square test. Univariate and multivariate analyses were performed to identify predictors of slow/no reflow phenomenon. Receiver operating characteristic (ROC) analysis was used to evaluate the sensitivity and specificity of SI predicting slow/no reflow. All p <0.05 were considered statistically significant. Analyses were done with statistical software SPSS 11.0 (SPSS, Inc., Chicago, IL).

## 3. Results

### 3.1. Clinical and Angiographic Characteristics

Among the 153 patients with AMI who underwent primary PCI, 29 patients underwent angiographic slow/no reflow during PCI. The prevalence of high thrombus burden (p=0.001) and in-hospital outcomes (p<0.001) were higher in patients with slow/no reflow than those with normal reflow. The SI was significantly different between patients with slow/no reflow and normal reflow (p=0.003). As a result, we found that the ratio of SI≥0.66 was much higher in slow/no reflow than that in normal reflow (p=0.001). Compared with the normal reflow group, the ratio of Killip classes 3 or 4 was higher in slow/no reflow group (p=0.034). Left ventricular ejection fraction (LVEF) after PCI was lower in patients with slow/no reflow (p=0.024), compared with patients with normal reflow. There was a definite association between preoperative usage of glycoprotein IIb/IIIa inhibitors, tirofiban, and initial TIMI level (Pearson's r=0.189,* p*=0.019), but preoperative treatment of tirofiban did not decrease the occurrence of slow/no reflow. The distribution of infarct-related coronary artery and cardiovascular risk factors including hypertension, hyperlipidemia, diabetes mellitus, and in-hospital medication were similar between these two groups (Table 1). In addition, the stent diameter and length, maximal balloon pressure, and aspiration ratio between these two groups were also similar.

### 3.2. Preoperative Laboratory Data of Patients

Plasma glucose (p<0.001), White blood cells (p=0.011), neutrophil counts (p=0.002), neutrophil to lymphocyte ratio (NLR) (p=0.012), hs-CRP (p=0.045), and pro-BNP (p=0.041) on admission were higher in patients with slow/no reflow than those with normal reflow. Other hematological parameters such as hemoglobin, platelet count, mean platelet volume (MPV), platelet distribution width (PDW), and platelets to lymphocytes ratio (PLR) were similar between these two groups. Furthermore, the levels of serum cTNI, creatinine, and urea nitrogen were of no statistical significance between patients with slow/no reflow and normal reflow (Table 2).

### 3.3. Predictors for Angiographic Slow/No Reflow

**Figure 1:**
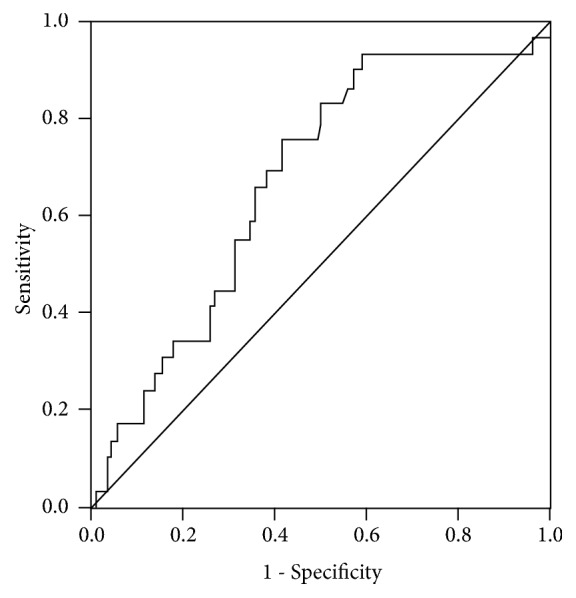
Receiver operating characteristic analysis displaying the power of shock index in the prediction of slow/no reflow phenomenon.

Univariate analysis showed that SI≥0.66 (p=0.001), high thrombus burden (p=0.002), Killip classes 3/4 (p=0.040), plasma glucose (p<0.001), White blood cells (p=0.014), neutrophil counts (p=0.004), neutrophil to lymphocyte ratio (NLR) (p=0.019), and hs-CRP (p=0.009) on admission were associated with lesion progression. After adjustment for age, sex, and smoking, multivariate logistic regression analysis revealed that SI≥0.66 (OR=3.645, 95%CI=1.206-11.018, and p=0.022), high thrombus burden (OR=3.536, 95%CI=1.324-9.438, and p=0.031), and plasma glucose (OR=1.116, 95%CI=1.010-1.232, and p=0.012) were still independent determinants for coronary slow/no reflow in patients with AMI undergoing emergent PCI (Table 3). ROC curve analysis revealed that the sensitivity was 76% and specificity was 59% for SI=0.66 to predict slow/no reflow phenomenon, and the area under the curve of SI was 0.672 (CI 95% 0.568–0.776,* p*=0.004) (Figure 1).

## 4. Discussion

The slow/no reflow phenomenon is frequently observed during the PCI, which can counteract partial benefits from catheter intervention. Although the exact mechanism of slow/no reflow was not fully clarified, inflammation, endothelial dysfunction, changes in blood components, reperfusion time, and angiographic characteristics of coronary lesions have been proposed in the pathogenesis of slow/no reflow [10–15, 21]. In this study, we found that independent predictors of angiographic slow/no reflow during the emergent PCI were SI≥ 0.66, plasma glucose on admission, and high thrombus burden on initial angiography.

In our current investigation, we found that high thrombus burden was associated with the phenomenon of slow/no reflow. In fact, the thrombus burden as a strong indicator for slow/no reflow has been reported in many studies [22, 23]. High thrombus burden was usually accompanied with the extended time to reperfusion, since delayed reperfusion leads to thrombus gathering more and more erythrocytes and becoming more steady; more importantly, red thrombi tend to form fragments and lead to distal embolization during the balloon dilatation [7, 24]. As a result, microcirculatory dysfunction aggravates myocardial reperfusion injury and is associated with a higher risk to slow/no reflow and adverse cardiac events. Intensified antiplatelet treatment significantly improves platelet aggregation and inhibits thrombosis formation [25]. In our study, the rate of preoperative use of tirofiban was similar between two groups. The use of tirofiban was not an independent indicator for slow/no reflow, which significantly improves the initial TIMI. The effects of preoperative treatment with tirofiban on the prognosis of acute coronary syndrome (ACS) patients were extensively investigated in the past two decades, but the results were inconsistent [15, 26]. Skyschally et al. reported that distal coronary embolization was associated with severe regional contractile dysfunction in animal model [27]; we also found that the value of LVEF after PCI was remarkably lower in patients with slow/no reflow than that in normal reflow. It is well known that LVEF was an independent predictor for adverse prognosis in patients with ACS [28].

Higher heart rate on admission was an important predictor for death or cardiac events in patients with ACS, and elevated resting heart rate was related to poor outcomes in heart failure with reduced ejection fraction and preserved ejection fraction [29, 30]. In addition, it was mentioned that higher heart rate was associated with no reflow in patients with STEMI following primary PCI [22]. Low blood pressure is closely related to no reflow and higher in-hospital mortality and poorer cardiac function [11, 20]. The concept of SI, defined as the ratio of heart rate to systolic blood pressure, is a relatively objective indicator independently of systolic blood pressure and heart rate under excitement of sympathetic nerve [31], which cause a concurrent increase of heart rate and systolic blood pressure. Studies showed that SI was strongly associated with in-hospital mortality in patients with ACS following primary PCI, and SI≥0.66 representing a cutoff value for clinical prediction was demonstrated in several studies [16, 17]. In the current study, we found that patients with slow/no reflow had higher ratio of SI≥0.66, compared with the normal reflow. After adjusting other risk factors, SI≥0.66 was still a pivotal predictor for slow/no reflow.

It has been mentioned that hyperglycemia was associated with an impairment of microvascular function and could cause angiographic slow/no reflow [32]. There are several mechanisms of hyperglycemia-associated angiographic slow/no reflow. Specially, hyperglycemia aggravates platelet-dependent thrombosis, inhibits endothelium dependent vasodilatation, and decreases collateral blood flow by decreasing nitric oxide availability [33–35]. In accordance with previous studies, multivariate logistic regression showed that the hyperglycemia on admission was an independent factor for slow/no reflow in our study.

Abnormality of hematologic parameters has also been implicated in the pathogenesis of the slow/no reflow phenomenon. Elevated WBC and NLR were reported as a risk factor for indicating systemic inflammatory response, and MPV as well as PDW was a potentially useful marker of platelet activity [22, 36]. In our study, we found that WBC, neutrophil, NLR, and hs-CRP were notably increased in slow/no reflow group than those in normal group. However, these inflammatory indicators were not an independent predictor factor for slow/no reflow. We supposed that it was possibly associated with our relatively small samples. Moreover, above parameters as a powerful predictor should be further verified in other studies.

The process of PCI itself was related to PCI-related slow/no reflow, and it was demonstrated that the length of implanted stent and overexpansion of stent were associated with occurrence of no reflow [37, 38]. The balloon or stent expansion crushes the plaques and causes the lipid core rupture; lipid fraction activates the thrombosis formation and fragment obstructs the distal microcirculation. It has been approved that there is a possible relationship between lipid-rich plaque and no reflow via preinterventional optical coherence tomography examination [39]. In the current study, we observed that balloon dilatation pressures of stenting were slightly higher in slow/no reflow group than those in normal group. However, the stent length and diameter were similar between these two groups.

In conclusion, considering the complicated mechanism and severe adverse prognosis of slow/no reflow phenomenon, it is very meaningful to find more useful and powerful predictors to prejudge the occurrence of slow/no reflow. In this retrospective investigation, we observed that plasma glucose, SI≥0.66 on admission, and high thrombus load on initial angiography were related to angiographic slow/no reflow phenomenon in patients with AMI following primary PCI. ROC curve analysis showed that the area under the curve of SI was 0.672. We considered that more than 40% patients in normal flow had SI≥0.66, which possibly affected the power of SI predicting slow/no reflow phenomenon. In addition, we are conscious that the sample size in our study was small; thus some group comparisons may have lacked power to detect significant differences for selected variables. In addition, larger-scale studies should be performed to confirm the association between above predictors and slow/no reflow.

## Figures and Tables

**Table 1:** Clinical and coronary angiographic features in patients with normal flow and slow/no reflow.

	Normal flow group	Slow/no reflow group	P value
	n=124	n=29	
Male gender, n (%)	96(77.4)	25(86.2)	0.295
Age, years	63±14	61±13	0.423
Cigarette smoking, n (%)	60(48.4)	16(55.2)	0.511
Heart rate	83±13	86±14	0.248
Systolic blood pressure, mmHg	129±20	123±18	0.159
Diastolic blood pressure, mmHg	80±14	84±19	0.306
Shock index	0.65±0.09	0.71±0.09	0.003
Shock index≥0.66, n (%)	51(41.1)	22(75.9)	0.001
Diabetes mellitus, n (%)	34(27.4)	8(27.6)	0.986
Hypertension, n (%)	65(52.4)	16(55.2)	0.789
Hyperlipidemia, n (%)	31(25.0)	10(34.5)	0.299
Previous cerebrovascular disease, n (%)	15(12.1)	5(17.2)	0.459
Time to reperfusion (hour)	7.63±5.90	7.52±5.65	0.926
STEMI, n (%)	84(67.7)	21(72.4)	0.625
Initial TIMI flow 0/1 grade, n (%)	95(76.6)	26(89.7)	0.120
High thrombus burden, n (%)	44(35.5)	20(69.0)	0.001
Preoperative use of tirofiban, n (%)	75(60.5)	17(58.6)	0.854
Aspiration, n (%)	24(19.4)	6(20.7)	0.871
Infarct-related coronary artery			0.357
LAD, n (%)	62(50.0)	15(51.7)	
RCA, n (%)	41(33.1)	12(41.4)	
LCA, n (%)	21(16.9)	2(6.9)	
Killip classification			0.034
1/2, n (%)	112(90.3)	22(75.9)	
3/4, n (%)	12(9.7)	7(24.1)	
Multivessel disease, n (%)	60(48.4)	15(51.7)	0.746
Stent diameter, mm	3.07±0.30	3.17±0.35	0.100
Stent length, mm	35±15	34±17	0.677
Maximal balloon pressure, atm	13±3	14±3	0.085
LVEF after PCI, %	54±9	49±8	0.024
In-hospital medication			
Aspirin, n (%)	123(99.2)	28(96.6)	0.259
Clopidogrel or ticagrelor, n (%)	123(99.2)	28(96.6)	0.259
ACEI or ARB, n (%)	74(59.7)	17(58.6)	0.917
*β*-Blockers, n (%)	54(43.5)	10(34.5)	0.373
Statins, n (%)	118(95.2)	27(93.1)	0.654
In-hospital outcomes, n (%)	2(1.6)	5(17.2)	<0.001

STEMI: ST-elevation myocardial infarction; TIMI: thrombolysis in myocardial infarction; LAD: left anterior descending artery; RCA: right coronary artery; LCA: left circumflex artery; LVEF: left ventricular ejection fraction; PCI: percutaneous coronary intervention; ACEI: angiotensin converting enzyme inhibitor; ARB: angiotensin-receptor blocker.

**Table 2:** Preoperative laboratory data of patients.

	Normal flow group	Slow/No reflow group	P value
	n=124	n=29	
Plasma glucose, mmol/l	8.57±3.93	12.52±6.86	<0.001
WBC (×10^∧^9 /L)	10.26±2.74	11.71±2.63	0.011
neutrophil (×10^∧^9 /L)	8.14±2.83	9.97±2.97	0.002
lymphocyte (×10^∧^9 /L)	1.58±1.01	1.22±0.83	0.082
NLR	7.24±6.20	10.41±5.38	0.012
Hemoglobin (g/L)	142±17	143±22	0.868
PLT (×10^∧^9 /L)	194±59	192±51	0.821
PLR	157±106	190±94	0.124
MPV, fL	9.77±1.28	10.16±1.47	0.151
PDW (%)	15.39±1.60	15.97±1.74	0.086
hs-CRP, mg/l	13.43±12.19	21.46±19.89	0.045
Blood urea nitrogen, mmol /L	6.31±2.39	5.99±1.85	0.501
Creatinine, *μ*mol/L	83.51±28.34	83.87±20.22	0.950
cTNI, *μ*g/L	15.65±28.35	19.85±25.17	0.465
pro-BNP, pg/mL	438±736	769±940	0.041

Data are mean ± SD.

WBC: white blood cell count; NLR: neutrophil to lymphocyte ratio; PLT: platelet count; PLR: platelets to lymphocytes ratio; MPV: mean platelet volume; PDW: platelet distribution width; hsCRP: high sensitivity C-reactive protein; cTNI: cardiac troponin I; pro-BNP: probrain natriuretic peptide.

**Table 3:** Multivariate analysis of risk factors for patients with normal flow and slow/no reflow.

Variables	OR	95%CI	p value
Plasma glucose	1.116	1.010-1.232	0.031
High thrombus burden	3.536	1.324-9.438	0.012
Shock index≥0.66	3.645	1.206-11.018	0.022

OR: odds ratio; CI: confidence internal.

## Data Availability

The data used to support the findings of this study are available from the corresponding author upon request.
